# Crystal structure of 4-bromo­anilinium 4-methyl­benzene­sulfonate

**DOI:** 10.1107/S2056989015002686

**Published:** 2015-02-13

**Authors:** P. K. Sivakumar, M. Krishna Kumar, R. Mohan Kumar, G. Chakkaravarthi, R. Kanagadurai

**Affiliations:** a Department of Physics, M. N. M. Jain Engineering College, Chennai 600 097, India; b Department of Physics, Presidency College, Chennai 600 005, India; c Department of Physics, CPCL Polytechnic College, Chennai 600 068, India

**Keywords:** crystal structure, anilinium, 4-methyl­benzene­sulfonate, hydrogen bonding

## Abstract

In the crystal of the title mol­ecular salt, C_6_H_7_BrN^+^·C_7_H_7_O_3_S^−^, the anions and cations are linked *via* N—H⋯O hydrogen bonds forming layers, enclosing *R*_2_^2^(4) ring motifs, lying parallel to (001). Within the layers there are short O⋯O contacts of 2.843 (2) Å.

## Related literature   

For the crystal structure of 4-chloro­anilinium 4-methyl­benzene­sulfonate, isostructural with the title salt, see: Jasinski *et al.* (2011 ▸). For the crystal structure of other 4-methyl­benzene­sulfonate salts, see, for example: Krishnakumar *et al.* (2012 ▸); Sudhahar *et al.* (2013 ▸).
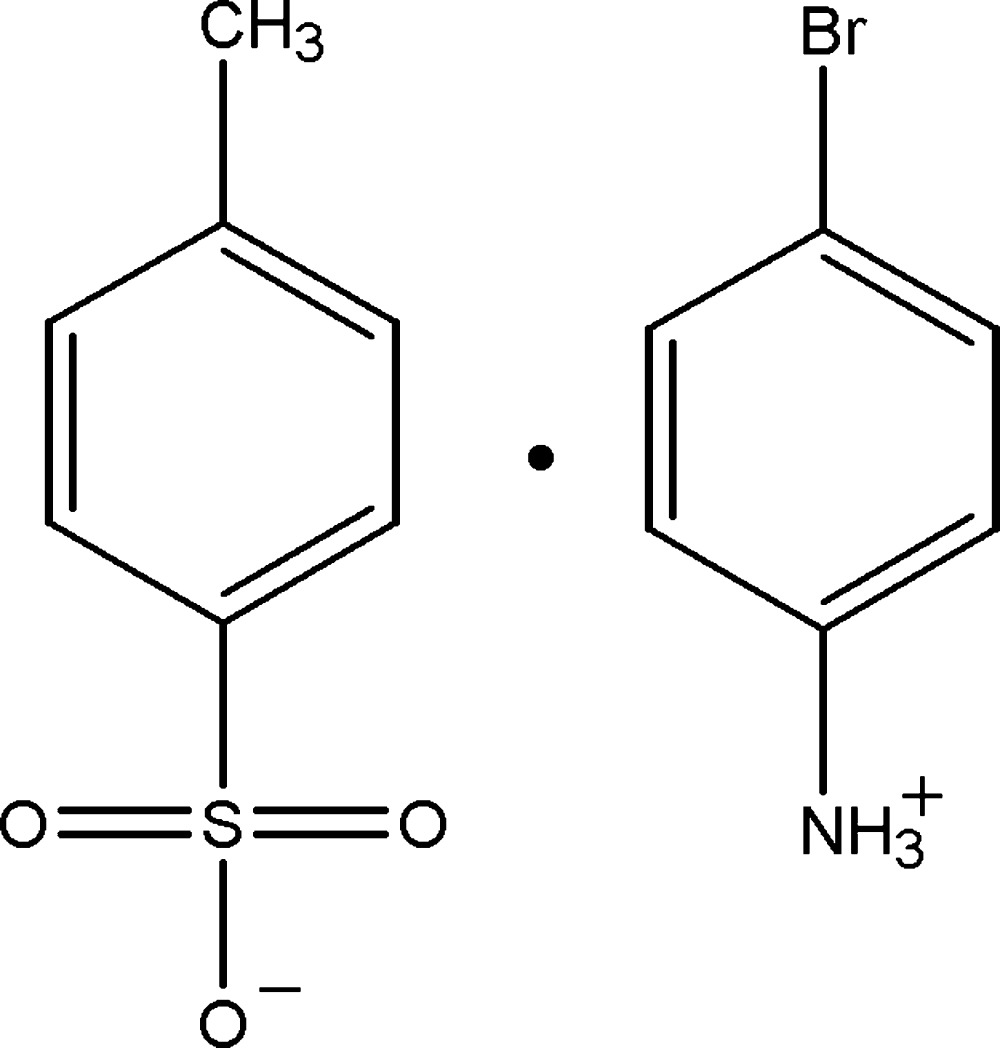


## Experimental   

### Crystal data  

C_6_H_7_BrN^+^·C_7_H_7_O_3_S^−^
*M* *_r_* = 344.22
Triclinic, 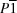

*a* = 5.7908 (3) Å
*b* = 7.6004 (4) Å
*c* = 15.9073 (7) Å
α = 94.716 (2)°
β = 96.520 (3)°
γ = 92.732 (2)°
*V* = 692.09 (6) Å^3^
*Z* = 2
Mo *K*α radiation
μ = 3.12 mm^−1^
*T* = 295 K
0.24 × 0.20 × 0.18 mm

### Data collection  

Bruker Kappa APEXII CCD diffractometer
Absorption correction: multi-scan (*SADABS*; Sheldrick, 1996 ▸) *T*_min_ = 0.521, *T*_max_ = 0.603
11503 measured reflections
3001 independent reflections
2526 reflections with *I* > 2σ(*I*)
*R*_int_ = 0.030

### Refinement  

*R*[*F* ^2^ > 2σ(*F* ^2^)] = 0.033
*wR*(*F* ^2^) = 0.083
*S* = 1.03
3001 reflections
175 parameters
H-atom parameters constrained
Δρ_max_ = 0.45 e Å^−3^
Δρ_min_ = −0.56 e Å^−3^

### 

Data collection: *APEX2* (Bruker, 2004 ▸); cell refinement: *SAINT* (Bruker, 2004 ▸); data reduction: *SAINT*; program(s) used to solve structure: *SHELXS97* (Sheldrick, 2008 ▸); program(s) used to refine structure: *SHELXL97* (Sheldrick, 2008 ▸); molecular graphics: *PLATON* (Spek, 2009 ▸); software used to prepare material for publication: *SHELXL97* and *PLATON* (Spek, 2009 ▸).

## Supplementary Material

Crystal structure: contains datablock(s) global, I. DOI: 10.1107/S2056989015002686/su5079sup1.cif

Structure factors: contains datablock(s) I. DOI: 10.1107/S2056989015002686/su5079Isup2.hkl

Supporting information file. DOI: 10.1107/S2056989015002686/su5079Isup3.cml

**Fig. 1.**
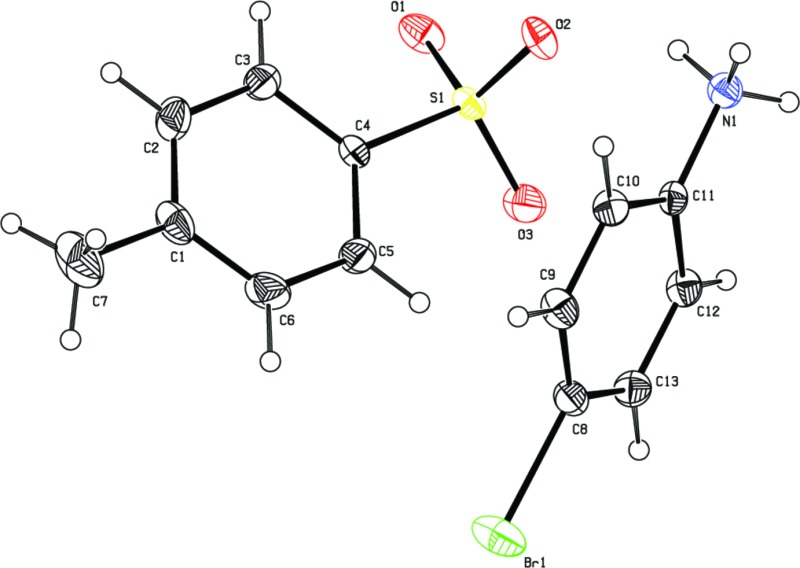
The molecular structure of the title molecular salt, with atom labelling. Displacement ellipsoids ae drawn at the 30% probability level.

**Fig. 2.**
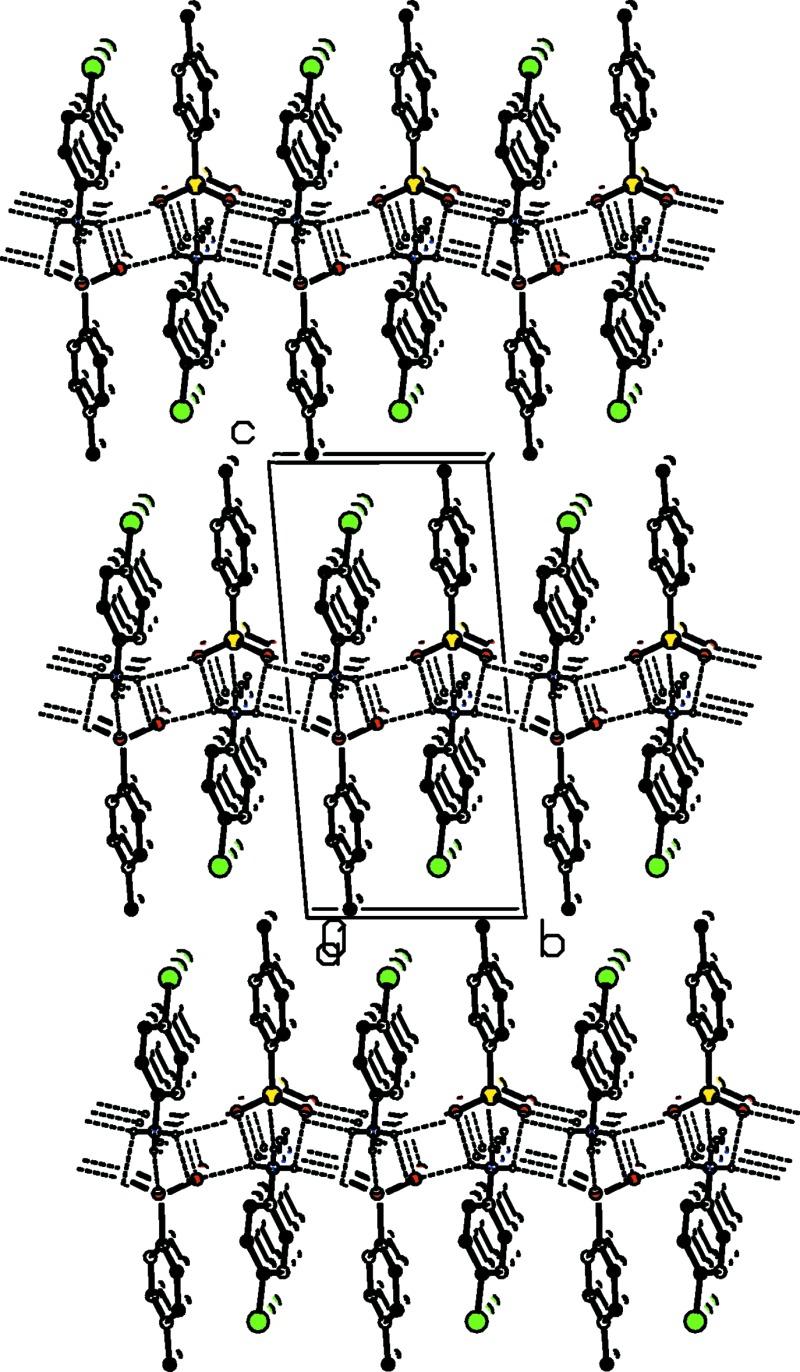
The crystal packing of the title molecular salt, viewed along the a axis. Hydrogen bonds are shown as dashed lines (see Table 1 for details; H atoms not involved in hydrogen bonding have been omitted for clarity).

. DOI: 10.1107/S2056989015002686/su5079fig1.tif
The mol­ecular structure of the title mol­ecular salt, with atom labelling. Displacement ellipsoids ae drawn at the 30% probability level.

a . DOI: 10.1107/S2056989015002686/su5079fig2.tif
The crystal packing of the title mol­ecular salt, viewed along the *a* axis. Hydrogen bonds are shown as dashed lines (see Table 1 for details; H atoms not involved in hydrogen bonding have been omitted for clarity).

CCDC reference: 1048164

Additional supporting information:  crystallographic information; 3D view; checkCIF report

## Figures and Tables

**Table 1:** Hydrogen-bond geometry (, )

*D*H*A*	*D*H	H*A*	*D* *A*	*D*H*A*
N1H1*A*O2	0.89	2.07	2.879(2)	151
N1H1*C*O1^i^	0.89	2.46	2.971(2)	117
N1H1*A*O2^i^	0.89	2.46	3.096(2)	129
N1H1*B*O3^ii^	0.89	1.91	2.794(2)	172
N1H1*C*O1^iii^	0.89	2.04	2.904(2)	165

Symmetry codes: (i) 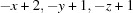; (ii) 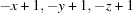; (iii) 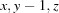.

## supplementary crystallographic information

### S1. Structural commentary

The molecular structure of the title salt is illustrated in Fig. 1. The bond lengths and angles are similar to those reported for the 4-chloro­anilinium analogue (Jasinski *et al.*, 2011), and for other similar 4-methyl­benzene­sulfonate salts (Krishnakumar *et al.*, 2012; Sudhahar *et al.*, 2013).

In the crystal, the benzene rings (C1—C6) and (C8—C13) make a dihedral angle of 50.89 (11) °. The cations and anions are linked via N—H···O hydrogen bonds which generates R_2_^2^(4) ring motifs, and form layers parallel to (001); see Table 1 and Fig. 2. Within the layers there are short O1···O1^i^ contacts of 2.843 (2) Å [symmetry code: (i) -x+2, -y+2, -z+1].

### S2. Synthesis and crystallization

The title compound was synthesized in ethanol by mixing 4-bromo­aniline (2.37 g) and *p*-toluene­sulfonic­acid (2.62 g) in an equimolar ratio. The saturated solution was allowed to evaporating slowly at room temperature. After a period of three weeks colourless block-like crystals appeared.

### S3. Refinement

Crystal data, data collection and structure refinement details are summarized in Table 2. H atoms were positioned geometrically and refined using a riding model: N—H = 0.89 Å, C—H = 0.93 - 96 Å with U_iso_(H) = 1.5U_eq_(N,C) for the ammonium and methyl H atoms and = 1.2U_eq_(C) for other H atoms.

### Crystal data

**Table:**

C_6_H_7_BrN^+^·C_7_H_7_O_3_S^−^	*Z* = 2
*M**_r_* = 344.22	*F*(000) = 3048
Triclinic, *P*1	*D*_x_ = 1.652 Mg m^−^^3^
Hall symbol: -P 1	Mo *K*α radiation, λ = 0.71073 Å
*a* = 5.7908 (3) Å	Cell parameters from 348 reflections
*b* = 7.6004 (4) Å	θ = 1.3–27.1°
*c* = 15.9073 (7) Å	µ = 3.12 mm^−^^1^
α = 94.716 (2)°	*T* = 295 K
β = 96.520 (3)°	Block, colourless
γ = 92.732 (2)°	0.24 × 0.20 × 0.18 mm
*V* = 692.09 (6) Å^3^	

### Data collection

**Table:**

Bruker Kappa APEXII CCD diffractometer	3001 independent reflections
Radiation source: fine-focus sealed tube	2526 reflections with *I* > 2σ(*I*)
Graphite monochromator	*R*_int_ = 0.030
ω and φ scan	θ_max_ = 27.1°, θ_min_ = 1.3°
Absorption correction: multi-scan (*SADABS*; Sheldrick, 1996)	*h* = −7→7
*T*_min_ = 0.521, *T*_max_ = 0.603	*k* = −9→9
11503 measured reflections	*l* = −20→20

### Refinement

**Table:**

Refinement on *F*^2^	Secondary atom site location: difference Fourier map
Least-squares matrix: full	Hydrogen site location: inferred from neighbouring sites
*R*[*F*^2^ > 2σ(*F*^2^)] = 0.033	H-atom parameters constrained
*wR*(*F*^2^) = 0.083	*w* = 1/[σ^2^(*F*_o_^2^) + (0.0406*P*)^2^ + 0.3371*P*] where *P* = (*F*_o_^2^ + 2*F*_c_^2^)/3
*S* = 1.03	(Δ/σ)_max_ < 0.001
3001 reflections	Δρ_max_ = 0.45 e Å^−^^3^
175 parameters	Δρ_min_ = −0.56 e Å^−^^3^
0 restraints	Extinction correction: *SHELXL97* (Sheldrick, 2008), Fc^*^=kFc[1+0.001xFc^2^λ^3^/sin(2θ)]^-1/4^
Primary atom site location: structure-invariant direct methods	Extinction coefficient: 0.034 (2)

### Special details

**Table:**

Geometry. All esds (except the esd in the dihedral angle between two l.s. planes) are estimated using the full covariance matrix. The cell esds are taken into account individually in the estimation of esds in distances, angles and torsion angles; correlations between esds in cell parameters are only used when they are defined by crystal symmetry. An approximate (isotropic) treatment of cell esds is used for estimating esds involving l.s. planes.
Refinement. Refinement of F^2^ against ALL reflections. The weighted R-factor wR and goodness of fit S are based on F^2^, conventional R-factors R are based on F, with F set to zero for negative F^2^. The threshold expression of F^2^ > 2sigma(F^2^) is used only for calculating R-factors(gt) etc. and is not relevant to the choice of reflections for refinement. R-factors based on F^2^ are statistically about twice as large as those based on F, and R- factors based on ALL data will be even larger.

### Fractional atomic coordinates and isotropic or equivalent isotropic displacement parameters (Å^2^)

**Table:**

	*x*	*y*	*z*	*U*_iso_*/*U*_eq_	
C1	1.0889 (4)	0.8014 (3)	0.89050 (15)	0.0401 (6)	
C2	1.2329 (4)	0.8603 (4)	0.83410 (17)	0.0428 (6)	
H2	1.3820	0.9068	0.8546	0.051*	
C3	1.1611 (4)	0.8518 (3)	0.74779 (15)	0.0353 (5)	
H3	1.2613	0.8909	0.7106	0.042*	
C4	0.9385 (3)	0.7845 (3)	0.71760 (12)	0.0233 (4)	
C5	0.7913 (4)	0.7260 (3)	0.77291 (15)	0.0351 (5)	
H5	0.6416	0.6807	0.7525	0.042*	
C6	0.8673 (4)	0.7348 (4)	0.85901 (15)	0.0439 (6)	
H6	0.7674	0.6952	0.8962	0.053*	
C7	1.1741 (6)	0.8053 (5)	0.98404 (18)	0.0686 (9)	
H7A	1.2377	0.6944	0.9958	0.103*	
H7B	1.0464	0.8251	1.0166	0.103*	
H7C	1.2922	0.8991	0.9992	0.103*	
C8	0.4302 (4)	0.3193 (3)	0.77075 (14)	0.0317 (5)	
C9	0.6357 (4)	0.2352 (3)	0.77106 (15)	0.0342 (5)	
H9	0.7066	0.1958	0.8209	0.041*	
C10	0.7353 (4)	0.2102 (3)	0.69649 (14)	0.0296 (5)	
H10	0.8749	0.1548	0.6958	0.036*	
C11	0.6259 (3)	0.2681 (3)	0.62306 (13)	0.0253 (4)	
C12	0.4203 (4)	0.3529 (3)	0.62302 (14)	0.0309 (5)	
H12	0.3492	0.3920	0.5731	0.037*	
C13	0.3209 (4)	0.3792 (3)	0.69759 (15)	0.0329 (5)	
H13	0.1826	0.4363	0.6986	0.039*	
N1	0.7352 (3)	0.2443 (2)	0.54491 (11)	0.0301 (4)	
H1A	0.8264	0.3397	0.5401	0.045*	
H1B	0.6256	0.2287	0.5006	0.045*	
H1C	0.8204	0.1499	0.5464	0.045*	
O1	0.9616 (3)	0.9187 (2)	0.57494 (10)	0.0387 (4)	
O2	0.9290 (3)	0.6031 (2)	0.57251 (10)	0.0372 (4)	
O3	0.5965 (3)	0.7733 (3)	0.59796 (10)	0.0460 (4)	
Br1	0.29548 (5)	0.35751 (4)	0.873294 (18)	0.05714 (14)	
S1	0.84823 (9)	0.76863 (7)	0.60718 (3)	0.02575 (14)	

### Atomic displacement parameters (Å^2^)

**Table:**

	*U*^11^	*U*^22^	*U*^33^	*U*^12^	*U*^13^	*U*^23^
C1	0.0476 (14)	0.0480 (14)	0.0238 (12)	0.0154 (11)	−0.0024 (10)	−0.0011 (10)
C2	0.0341 (12)	0.0518 (15)	0.0382 (14)	0.0012 (11)	−0.0083 (10)	−0.0035 (11)
C3	0.0298 (11)	0.0428 (13)	0.0327 (12)	−0.0022 (9)	0.0030 (9)	0.0032 (10)
C4	0.0287 (10)	0.0214 (9)	0.0200 (10)	0.0048 (8)	0.0029 (7)	0.0010 (8)
C5	0.0315 (11)	0.0463 (14)	0.0273 (12)	−0.0034 (10)	0.0038 (9)	0.0050 (10)
C6	0.0460 (14)	0.0625 (17)	0.0261 (12)	0.0034 (12)	0.0111 (10)	0.0106 (11)
C7	0.077 (2)	0.098 (3)	0.0278 (14)	0.0243 (19)	−0.0093 (14)	−0.0014 (16)
C8	0.0353 (11)	0.0310 (11)	0.0287 (12)	0.0018 (9)	0.0061 (9)	−0.0007 (9)
C9	0.0399 (12)	0.0358 (12)	0.0267 (11)	0.0083 (10)	−0.0007 (9)	0.0055 (9)
C10	0.0289 (10)	0.0283 (11)	0.0315 (12)	0.0075 (8)	0.0003 (8)	0.0034 (9)
C11	0.0263 (10)	0.0220 (10)	0.0262 (11)	−0.0013 (8)	0.0009 (8)	−0.0012 (8)
C12	0.0300 (11)	0.0321 (11)	0.0299 (12)	0.0054 (9)	−0.0020 (9)	0.0046 (9)
C13	0.0273 (11)	0.0333 (12)	0.0379 (13)	0.0070 (9)	0.0020 (9)	0.0015 (10)
N1	0.0298 (9)	0.0316 (9)	0.0286 (10)	0.0032 (7)	0.0019 (7)	0.0029 (8)
O1	0.0566 (10)	0.0325 (8)	0.0315 (9)	0.0091 (7)	0.0160 (7)	0.0109 (7)
O2	0.0531 (10)	0.0281 (8)	0.0297 (9)	0.0057 (7)	0.0065 (7)	−0.0053 (7)
O3	0.0321 (9)	0.0787 (13)	0.0263 (9)	0.0106 (8)	−0.0017 (7)	0.0025 (8)
Br1	0.0649 (2)	0.0722 (2)	0.03948 (19)	0.02182 (15)	0.02132 (13)	0.00502 (14)
S1	0.0313 (3)	0.0287 (3)	0.0179 (3)	0.0066 (2)	0.00382 (19)	0.0019 (2)

### Geometric parameters (Å, º)

**Table:**

C1—C2	1.379 (4)	C8—Br1	1.894 (2)
C1—C6	1.380 (4)	C9—C10	1.380 (3)
C1—C7	1.511 (3)	C9—H9	0.9300
C2—C3	1.384 (3)	C10—C11	1.379 (3)
C2—H2	0.9300	C10—H10	0.9300
C3—C4	1.382 (3)	C11—C12	1.381 (3)
C3—H3	0.9300	C11—N1	1.460 (3)
C4—C5	1.377 (3)	C12—C13	1.380 (3)
C4—S1	1.767 (2)	C12—H12	0.9300
C5—C6	1.385 (3)	C13—H13	0.9300
C5—H5	0.9300	N1—H1A	0.8900
C6—H6	0.9300	N1—H1B	0.8900
C7—H7A	0.9600	N1—H1C	0.8900
C7—H7B	0.9600	O1—S1	1.4480 (16)
C7—H7C	0.9600	O2—S1	1.4513 (16)
C8—C9	1.377 (3)	O3—S1	1.4509 (17)
C8—C13	1.382 (3)		
			
C2—C1—C6	118.3 (2)	C8—C9—C10	119.2 (2)
C2—C1—C7	120.7 (3)	C8—C9—H9	120.4
C6—C1—C7	120.9 (3)	C10—C9—H9	120.4
C1—C2—C3	121.6 (2)	C11—C10—C9	119.39 (19)
C1—C2—H2	119.2	C11—C10—H10	120.3
C3—C2—H2	119.2	C9—C10—H10	120.3
C4—C3—C2	119.1 (2)	C10—C11—C12	121.2 (2)
C4—C3—H3	120.5	C10—C11—N1	118.94 (18)
C2—C3—H3	120.5	C12—C11—N1	119.80 (18)
C5—C4—C3	120.20 (19)	C13—C12—C11	119.56 (19)
C5—C4—S1	120.52 (16)	C13—C12—H12	120.2
C3—C4—S1	119.26 (16)	C11—C12—H12	120.2
C4—C5—C6	119.7 (2)	C12—C13—C8	118.9 (2)
C4—C5—H5	120.1	C12—C13—H13	120.5
C6—C5—H5	120.1	C8—C13—H13	120.5
C1—C6—C5	121.0 (2)	C11—N1—H1A	109.5
C1—C6—H6	119.5	C11—N1—H1B	109.5
C5—C6—H6	119.5	H1A—N1—H1B	109.5
C1—C7—H7A	109.5	C11—N1—H1C	109.5
C1—C7—H7B	109.5	H1A—N1—H1C	109.5
H7A—C7—H7B	109.5	H1B—N1—H1C	109.5
C1—C7—H7C	109.5	O1—S1—O3	113.04 (11)
H7A—C7—H7C	109.5	O1—S1—O2	111.33 (10)
H7B—C7—H7C	109.5	O3—S1—O2	113.23 (11)
C9—C8—C13	121.7 (2)	O1—S1—C4	106.17 (10)
C9—C8—Br1	119.41 (16)	O3—S1—C4	106.11 (10)
C13—C8—Br1	118.93 (17)	O2—S1—C4	106.31 (9)
			
C6—C1—C2—C3	−0.8 (4)	C9—C10—C11—C12	0.9 (3)
C7—C1—C2—C3	177.6 (3)	C9—C10—C11—N1	178.73 (19)
C1—C2—C3—C4	0.7 (4)	C10—C11—C12—C13	−0.5 (3)
C2—C3—C4—C5	−0.3 (3)	N1—C11—C12—C13	−178.31 (19)
C2—C3—C4—S1	−178.54 (19)	C11—C12—C13—C8	−0.1 (3)
C3—C4—C5—C6	0.0 (3)	C9—C8—C13—C12	0.4 (3)
S1—C4—C5—C6	178.18 (19)	Br1—C8—C13—C12	179.35 (16)
C2—C1—C6—C5	0.4 (4)	C5—C4—S1—O1	147.56 (18)
C7—C1—C6—C5	−178.0 (3)	C3—C4—S1—O1	−34.23 (19)
C4—C5—C6—C1	0.0 (4)	C5—C4—S1—O3	27.0 (2)
C13—C8—C9—C10	0.0 (3)	C3—C4—S1—O3	−154.76 (18)
Br1—C8—C9—C10	−178.95 (17)	C5—C4—S1—O2	−93.79 (19)
C8—C9—C10—C11	−0.7 (3)	C3—C4—S1—O2	84.42 (19)

### Hydrogen-bond geometry (Å, º)

**Table:**

*D*—H···*A*	*D*—H	H···*A*	*D*···*A*	*D*—H···*A*
N1—H1*A*···O2	0.89	2.07	2.879 (2)	151
N1—H1*C*···O1^i^	0.89	2.46	2.971 (2)	117
N1—H1*A*···O2^i^	0.89	2.46	3.096 (2)	129
N1—H1*B*···O3^ii^	0.89	1.91	2.794 (2)	172
N1—H1*C*···O1^iii^	0.89	2.04	2.904 (2)	165

Symmetry codes: (i) −*x*+2, −*y*+1, −*z*+1; (ii) −*x*+1, −*y*+1, −*z*+1; (iii) *x*, *y*−1, *z*.
