# Supplementary figures and images for: Crystal structure of 4-bromo­anilinium 4-methyl­benzene­sulfonate
Source: Acta Crystallogr E Crystallogr Commun. 2015 Feb 13;71(Pt 3):o163–4. doi: 10.1107/S2056989015002686 (PMC4350755; doi:10.1107/S2056989015002686)

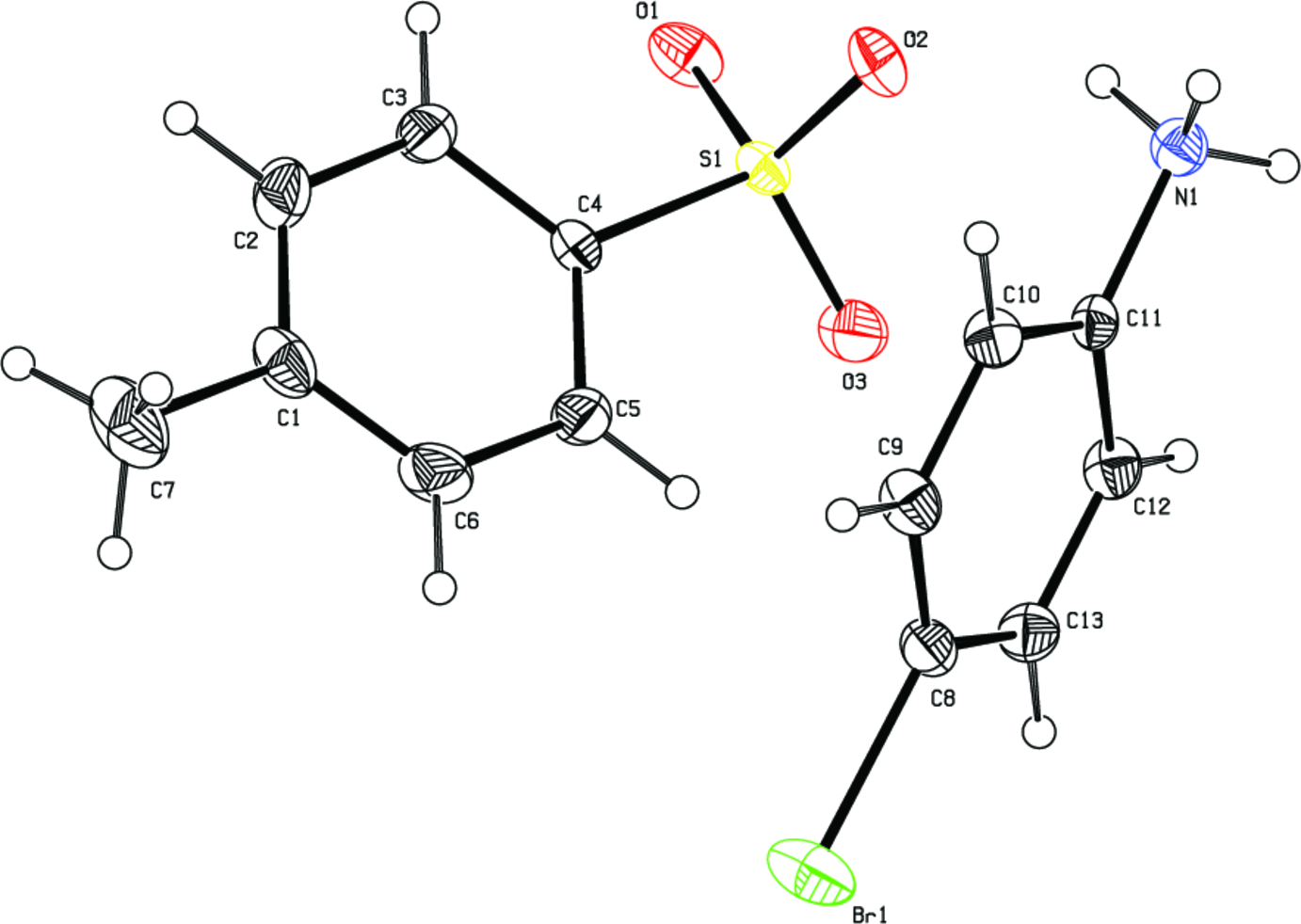

Supplement: Supplementary file 4 [file e-71-0o163-fig1.tif]

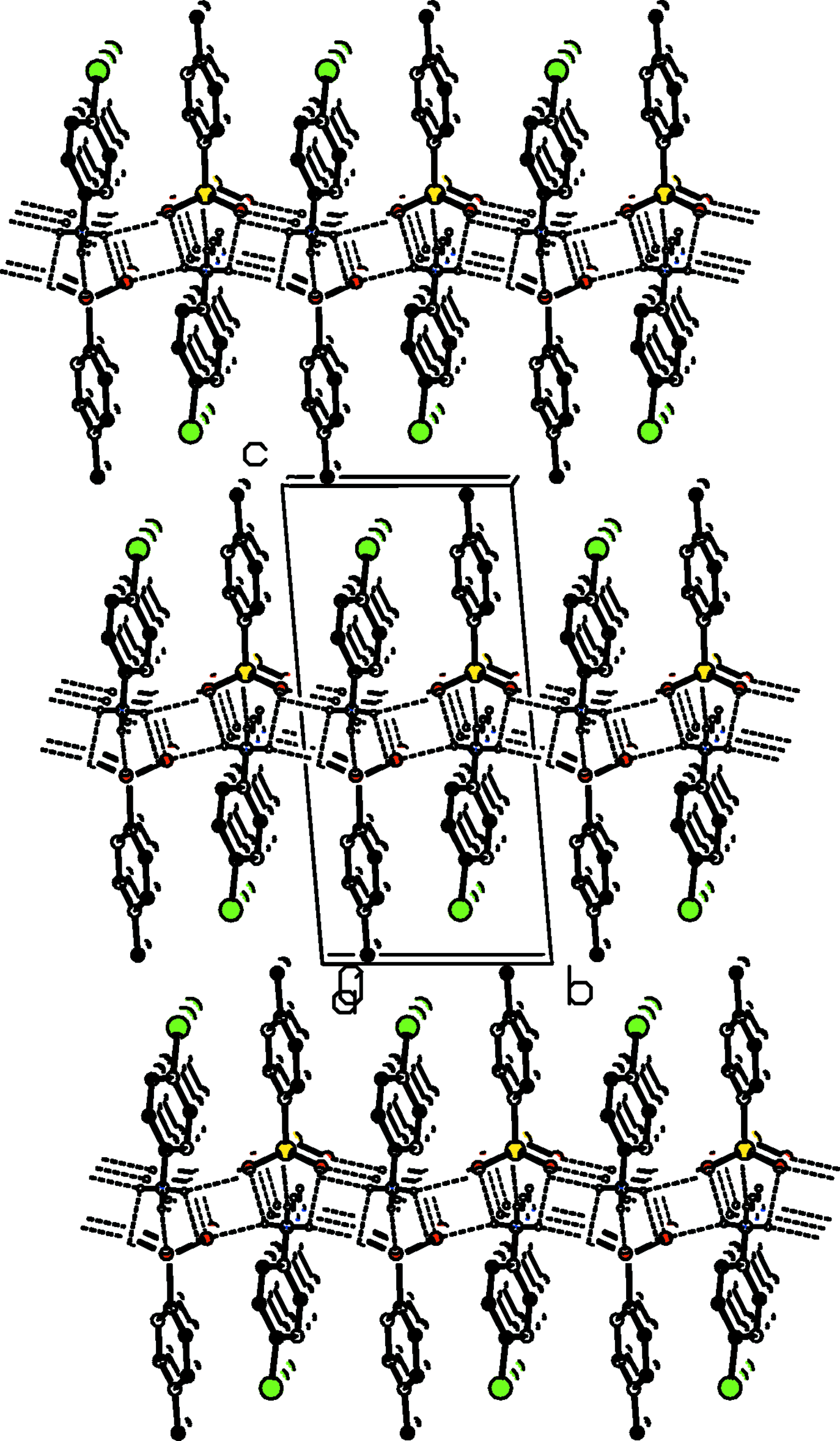

Supplement: Supplementary file 5 [file e-71-0o163-fig2.tif]
